# The impact of epidural analgesia compared to systemic opioid-based analgesia with regard to length of hospital stay and recovery of bowel function: retrospective evaluation of 1555 patients undergoing thoracotomy

**DOI:** 10.1186/s13019-014-0175-8

**Published:** 2014-11-23

**Authors:** Sandra Kampe, Gerhard Weinreich, Christopher Darr, Kolja Eicker, Georgios Stamatis, Thomas Hachenberg

**Affiliations:** Department of Anesthesiology and Pain Medicine, Ruhrlandklinik, West German Lung Center – University Hospital Essen, University Duisburg-Essen, Tüschener Weg 40, Essen, 45239 Germany; Department of Pneumology, Ruhrlandklinik, West German Lung Center, University Hospital Essen, University Duisburg-Essen, Essen, Germany; Department of Thoracic Surgery and Thoracic Endoscopy, Ruhrlandklinik, West German Lung Center – University Hospital Essen, University Duisburg-Essen, Essen, Germany; Department of Anesthesiology and Intensive Care Medicine, University Hospital Magdeburg, Otto von Guericke University Magdeburg, Magdeburg, Germany

**Keywords:** Major thoracic surgery, Postoperative analgesia, Epidural analgesia, Oxycodone, Hospital stay, Bowel function

## Abstract

**Background:**

To assess the protocols of epidural analgesia versus systemic opioid-based analgesia retrospectively in 1555 thoracotomies in our thoracic centre during 2011–2013.

**Methods:**

Pain therapy is aggressive and standardized in our thoracic centre thoughout the complete postoperative stay. Patients receive either standardized epidural analgesia with ropivacaine + sufentanil 4–8 mls/h (500 mls bag) and are bridged when the epidural bag is finished to a standardized controlled-release oxycodone protocol with non opioid every 6 hours (EDA Group), or patients receive immediately postoperative standardized oral analgesic protocol with controlled-released oxycodone and non opioid every 6 h (Opioid Group). All patients are visited daily by a pain specialist throughout the whole stay.

**Results:**

Data of 1555 thoracotomies from 2011-2013 were analysed, 838 patients in the EDA Group and 717 patients in the Opioid Group. There was no difference with regard to sex or age between groups. 7.5% of patients in the EDA Group and 13% in the Oxy Group had a preexisting pain therapy (p = 0.001). In the EDA Group epidural analgesia was performed for 4.6 ± 1.5 days. Length of hospital stay was the same in both groups (EDA: 9.9.6 ± 4.9 vs Opioid: 9.6 ± 5.8 days). 84.7% of patients in the EDA Group and 79.1% of patients of the Oxy Group were dismissed with oral opioid (p < 0.004). When patients were dismissed with opioid medication patients in the EDA Group were dismissed with higher oxycodone opioid doses than patients in the Opioid Group (29.5 ± 15.2 mg vs 26.9 ± 15.2 mg, p = 0.01). There was no difference with regard to dejection time between the two groups (EDA: 3.8 ± 2.2 days vs Opioid: 3.7 ± 1.6 days, n.s.).

**Conclusion:**

We first present data monitoring postoperative analgesic protocols after thoracotomies throughout the whole stay in hospital until dismission. Our retrospective data indicate that patients with epidural analgesia stay as long in hospital as patients with systemic opioid based therapy. Patients with initial epidural analgesia are dismissed with higher oxycodone opioid doses than patients with initial opioid based postoperative analgesia. We found no difference in recovery of bowel function.

**Study limitations:**

The study design is retrospectively and results might be biased.

## Background

Pain after thoracic surgery is supposed to be one of the most recognized pain syndrome associated with a specific procedure [1]. To date regional anesthesia is recommended for postoperative analgesia after thoracotomy [1]-[4]. Besides many advantages, such as positive influence on postoperative pulmonary morbidity [5], postoperative epidural analgesia after thoracotomy is supposed to faster postoperative recovery [6],[7] and earlier restitution of bowel function than with opioid-based systemic postoperative analgesia [8],[9]. We recently showed that an aggressive systemic pain therapy can be very effective after anteroaxillary thoracotomy [10] and that epidural analgesia is not superior to systemic pain therapy with regard to establishing neuropathic or chronic postthoracotomy pain [11].

Pain therapy is consequent and standardized in our thoracic centre thoughout the complete postoperative stay. Patients receive either standardized epidural analgesia and are bridged when the epidural bag is finished to a standardized controlled-release oxycodone protocol, or patients receive immediately postoperative standardized oral analgesic protocol with controlled-released oxycodone and non opioid every 6 h. All patients are visited daily by a pain specialist throughout the whole stay.

The purpose of the present study was to assess the protocols of epidural analgesia and systemic analgesia retrospectively in 1555 thoracotomies during 2011-2013 with regard to length of postoperative hospital stay, recovery of bowel function and opioid dose on dismission from hospital.

## Methods

After obtaining local research committee approval (University of Essen, Chairperson Prof. Havers) we investigated the postoperative pain therapy protocols of our thoracotomy patients during January 2011 until March 2013.

All patients underwent thoracotomy in balanced or total intravenous anesthesia. The serratus muscle was not cut, but spread. At the end of surgery two 24 CH soft chest tubes were placed and the thoracic cavum was closed with three pericostal sutures.

Since 2009 pain therapy is standardized in our thoracic center. If a thoracic epidural catheter (Braun Melsungen^R^) is inserted, it is placed before induction of anesthesia at thoracic level 5/6 or 6/7. After administering the test dose of 3 mls bupivacaine 0.25% the continuous epidural infusion is commenced at 4-8 mls/h (EDA Group). The standard epidural drug combination is ropivacaine 0.2% +0.75 μg/ml sufentanil (500 mls bag).

Half an hour before the end of the surgical procedure all patients receive 2.5 g of IV dipyrone. Dipyrone is a non-opioid analgesic frequently used in the perioperative setting in Germany. After extubating the trachea patients without epidural catheter receive IV piritramide at the discretion of the anesthetist (Opioid Group). Piritramide is an opioid commonly used in Germany with approximately 0.7 times the potency of morphine. Patients are admitted to the intensive care unit for one night and receive 1 g of dipyrone every 6 hours and IV piritramide bolus doses when NRS (numeric rating scale, 0 = no pain up to 10 = worst pain imaginable) is >3. If patients have epidurals, the EDA is continued at 4-6 mls/h. If epidural analgesia is not effective, patients are switched to systemic analgesia before dismission from intensive care unit.

After discharge from the intensive care unit the systemic pain therapy patients receive controlled-release oxycodone/oxycodone with naloxone (Opioid Group) 30-0-20 mg, and from the 2nd day on ward 20-0-10 mg and IV dipyrone 1 g every 6 h. If the NRS is >4 IV rescue medication (IV parecoxib 40 mg) is administered. On the ward continuous epidural analgesia is continued with the 500 mls ropivacaine 0.2% +0.75 μg/ml sufentanil bag (EDA Group) and IV dipyrone 1 g every 6 h. On the second day on ward IV dipyrone 1 g is oralized to 0.5 g dipyrone 4 times daily. The epidurals are continued on 4-8 mls/h until the 500 mls bag is finished and patients are then patients are bridged to a standardized controlled-release oxycodone protocol with controlled-release oxycodone/oxycodone with naloxone 20-0-10 mg per day and oral dipyrone.

During their stay in hospital all patients are visited daily by a pain specialized anesthetist and pain medication is adjusted to the state of recovery. Moreover numeric rating scale (NRS) at rest and on coughing are documented by the nurses on the wards every 6 h, implicating that the rescue medication (parecoxib 40 mg IV) has been administered by the nurse when NRS scores >3 at rest and >5 on coughing. Thirty min after administration of rescue medication the NRS has to be re-assessed. If pain score is again >5 on coughing, the nurse has to contact the anesthetist responsible for postoperative pain therapy.

It is standardized in our thoracic centre that all patients (both analgesic regimens) receive macrogel 13 g three times a day orally from day 1 on ward.

We assessed our 2 analgesic protocols retrospectively for 1555 thoracotomies from 2011-2013. Surgeons decide on the time of discharge of patients from our hospital.

All variables were analyzed by methods of descriptive statistics (frequency, mean ± standard deviation, range). Differences between groups were analyzed by using the Mann-Whitney-*U* test and differences in proportions were statistically evaluated by using chi-square test. Statistical significance was determined at the p < 0.05 level. Statistical analysis was performed by using SPSS 20.0 statistical package.

## Results

Data of 1555 major thoracotomies from 2011-2013 were analyzed, 838 patients in the EDA Group and 717 patients in the Opioid Group (Table 1). In the Opioid Group 414 patients received controlled-release oxycodone alone, and 303 patients received controlled-release oxycodone combined with naloxone. The 1555 thoracotomies were in detail 46 pneumonectomies (Table 2), 28 bilobectomies (Table 3), 562 lobectomies (Table 4), 213 segmental resections (Table 5), 459 wedge resections (Table 6), 21 lung volume reductions (Table 7), 60 pleurectomies (Table 8), and 176 various procedures (Table 9). There was no difference with regard to sex or age between the EDA and the Opioid Group (Table 1) except the patient population undergoing pleurectomy (Table 8). More male patients received a pleurectomy than female patients (90% vs 60%, p = 0.007, Table 8). Within the 1555 thoracotomies more patients in the Opioid Group had a preexisting pain therapy than in the EDA Group (EDA: 7.5% vs Opioid Group13%, p < 0.001. Table 1). Regarding the bilobectomy procedure more patients in the EDA Group had a pre-existing pain therapy than patients in the Opioid Group (100% vs. 30%, p < 0.04, Table 3) and regarding the lobectomy procedure, more patients in the Opioid Group than in the EDA Group had a pre-existing pain therapy (13.8% vs 6.4%, p = 0.004, Table 4). In the EDA Group epidural analgesia was performed for 4.6 ± 1.5 days (Table 1). Hospital stay was the same in both groups (EDA: 9.9 ± 4.9 vs Oxy: 9.6 ± 5.8 days, not significant, Table 1). Only in patients undergoing pleurectomy hospital stay was shorter in the EDA Group (EDA Group: 10.8 ± 5.1 vs Opioid Group 13.3 ± 8.4 days, p < 0.01, Table 8). 84.7% of patients in the EDA Group and 79.1% of patients of the Opioid Group were dismissed with oral opioid (p < 0.004, Table 1), and when patients were dismissed with opioid medication patients in the EDA Group were dismissed with higher oxycodone opioid doses than patients in the Opioid Group (29.5 ± 15.2 mg vs 26.9 ± 15.2 mg, p = 0.01, Table 1). There was no difference with regard to recovery of bowel function between the two groups (EDA: 3.8 ± 2.2 days vs Oxy: 3.7 ± 1.6 days, n.s.).

**Table 1:** **Characteristics of patients undergoing thoracotomy (n = 1555)**

	EDA group (n = 838)	Opioid group (n = 717)	p-value
Age (years)	61.1 ± 13.6	61.0 ± 12.2	n.s.
Male gender (%)	57.9	60.4	n.s.
Hospital stay (days)	9.9 ± 4.9	9.6 ± 5.8	n.s.
Pre-existing pain therapy (%)	7.5	13.0	<0.001
Dismission with oral opioid (%)	84.7	79.1	0.004
Opiod dose after dismission (mg)	29.3 ± 15.2	26.9 ± 15.2	0.01
Time to first dejection (days)	3.8 ± 2.2	3.7 ± 1.6	n.s.
EDA duration (days)	4.6 ± 1.5	-	

Data are presented as means ± SD.

**Table 2:** **Characteristics of patients undergoing pneumectomy (n = 46)**

	EDA group (n = 32)	Opioid group (n = 14)	p-value
Age (years)	61.1 ± 13.6	58.1 ± 16.1	n.s.
Male gender (%)	68.8	78.6	n.s.
Hospital stay (days)	13.7 ± 5.8	14.1 ± 5.8	n.s.
Pre-existing pain therapy (%)	9.4	14.3	n.s.
Dismission with oral opioid (%)	81.3	57.1	n.s.
Opiod dose after dismission (mg)	29.0 ± 16.3	27.5 ± 18.5	n.s.
Time to first dejection (days)	4.6 ± 2.2	4.4. ± 1.9	n.s
EDA duration (days)	4.7 ± 1.6	-	

Data are presented as means ± SD.

**Table 3:** **Characteristics of patients undergoing bilobectomy (n = 28)**

	EDA group (n = 18)	Opioid group (n = 10)	p-value
Age (years)	59.6 ± 16.7	53.6 ± 11.8	n.s.
Male gender (%)	61.1	50.0	n.s.
Hospital stay (days)	10.8 ± 3.5	9.1 ± 2.4	n.s.
Pre-existing pain therapy (%)	100	30	0.04
Dismission with oral opioid (%)	72.2	90.0	n.s.
Opiod dose after dismission (mg)	24.2 ± 12.6	32.2 ± 16.2	n.s.
Time to first dejection (days)	4.1 ± 1.6	4.6 ± 1.7	n.s.
EDA duration (days)	4.9 ± 1.4	-	

Data are presented as means ± SD.

**Table 4:** **Characteristics of patients undergoing lobectomy (n = 562)**

	EDA group (n = 359)	Opioid group (n = 203)	p-value
Age (years)	62.1 ± 11.0	64.2 ± 10.4	0.02
Male gender (%)	58.5	54.2	n.s.
Hospital stay (days)	10.3 ± 4.9	10.6 ± 5.1	n.s.
Pre-existing pain therapy (%)	6.4	13.8	0.004
Dismission with oral opioid (%)	87.7	75.4	<0.001
Opiod dose after dismission (mg)	28.2 ± 14.5	24.7 ± 15.1	0.02
Time to first dejection (days)	3.8 ± 1.6	4.2 ± 2.7	0.06
EDA duration (days)	4.3 ± 1.5	-	

Data are presented as means ± SD.

**Table 5:** **Characteristics of patients undergoing segmental resection (n = 213)**

	EDA group (n = 113)	Opioid group (n = 100)	p-value
Age (years)	63.1 ± 11.6	62.5 ± 12.4	n.s.
Male gender (%)	56.6	51.0	n.s.
Hospital stay (days)	9.6 ± 4.4	8.9 ± 4.0	n.s.
Pre-existing pain therapy (%)	11.5	15.0	n.s.
Dismission with oral opioid (%)	84.1	82.0	n.s.
Opiod dose after dismission (mg)	28.8 ± 15.3	27.2 ± 14.7	n.s.
Time to first dejection (days)	3.5 ± 1.6	3.6 ± 1.9	n.s.
EDA duration (days)	4.3 ± 1.3	-	

Data are presented as means ± SD.

**Table 6:** **Characteristics of patients undergoing wedge excision, metastasectomy, bullectomy, and tumor extirpation (n = 459)**

	EDA group (n = 206)	Opioid group (n = 253)	p-value
Age (years)	58.7 ± 13.3	58.9 ± 15.1	n.s.
Male gender (%)	61.2	60.1	n.s.
Hospital stay (days)	8.4 ± 4.0	7.8 ± 3.9	0.09
Pre-existing pain therapy (%)	6.8	11.9	0.08
Dismission with oral opioid (%)	86.4	84.6	n.s.
Opiod dose after dismission (mg)	32.4 ± 14.5	27.8 ± 14.8	0.002
Time to first dejection (days)	3.4 ± 1.6	3.4 ± 1.5	n.s.
EDA duration (days)	4.1 ± 1.3	-	

Data are presented as means ± SD.

**Table 7:** **Characteristics of patients undergoing LVR - lung volume reduction - (n = 21)**

	EDA group (n = 12)	Opioid group (n = 9)	p-value
Age (years)	61.3 ± 9.2	56.7 ± 5.5	n.s.
Male gender (%)	50.0	33.3	n.s.
Hospital stay (days)	14.9 ± 5.6	15.8 ± 5.1	n.s.
Pre-existing pain therapy (%)	8.3	22.2	n.s.
Dismission with oral opioid (%)	83.3	88.9	n.s.
Opiod dose after dismission (mg)	28.5 ± 18.1	18.1 ± 10.3	n.s.
Time to first dejection (days)	4.6 ± 2.1	5.8 ± 2.6	n.s.
EDA duration (days)	4.5 ± 1.1	-	

Data are presented as means ± SD.

**Table 8:** **Characteristics of patients undergoing pleurectomy (n = 60)**

	EDA group (n = 30)	Opioid group (n = 30)	p-value
Age (years)	66.1 ± 8.2	59.8 ± 14.3	n.s.
Male gender (%)	90.0	60.0	0.007
Hospital stay (days)	10.8 ± 5.1	13.3 ± 8.4	<0.01
Pre-existing pain therapy (%)	10.0	13.3	n.s.
Dismission with oral opioid (%)	76.7	60.0	n.s.
Opiod dose after dismission (mg)	30.7 ± 15.7	28.9 ± 18.2	n.s
Time to first dejection (days)	3.2 ± 1.1	3.7 ± 3.0	n.s.
EDA duration (days)	4.4 ± 1.4	-	

Data are presented as means ± SD.

**Table 9:** **Characteristics of patients undergoing various procedures (n = 166)**

	EDA group (n = 68)	Opioid group (n = 98)	p-value
Age (years)	59.3 ± 16.0	59.9 ± 15.3	n.s.
Male gender (%)	58.8	66.3	n.s.
Hospital stay (days)	9.8 ± 5.7	11.1 ± 9.4	n.s.
Pre-existing pain therapy (%)	8.8	9.2	n.s.
Dismission with oral opioid (%)	73.5	76.5	n.s.
Opiod dose after dismission (mg)	27.7 ± 15.2	28.3 ± 16.4	n.s.
Time to first dejection (days)	3.5 ± 1.8	3.9 ± 2.2	n.s.
EDA duration (days)	4.5 ± 2.0	-	

Data are presented as means ± SD.

## Discussion

To our knowledge up to now no data are available with regard to postoperative recovery until dismission after having undergone thoracotomy. We retrospectively compared our two analgesic protocols in 1555 patients who were visited daily from a pain specialist and found no difference in recovery of bowel function (time of first dejection). First time of dejection was overall the fourth postoperative day. Epidural analgesia is supposed to earlier restitution of bowel function than opioid-based systemic postoperative analgesia [8],[9]. However, these systematic review data are based on data on abdominal surgery. Our data cannot confirm the superiority of thoracic epidural analgesia compared to postoperative opioids regarding bowel function. To our knowledge, no data of randomized controlled trials comparing epidurals and opioids on postoperative recovery after thoracotomy are available up to now. Our retrospective data indicate that patients with epidural analgesia stay as long in hospital as patients with initial systemic opioid-based therapy. There is only a mild tendency to a shorter hospital stay in the epidural group in patients with “smaller” surgical procedures, e.g. in the metastasectomy subgroup or in the pleurectomy subgroup. In all other surgical procedures such as lobectomies, bilobectomies, segmental resections with a greater resection of parenchyma no difference in hospital stay could be detected, although epidural analgesia in general is supposed to “catalyze” recovery [6],[7]. We cannot confirm these data. Again the current evicence is not based on randomized controlled trials. We suppose that the most influencing factor on postoperative recovery is, assuming that effective postoperative analgesia is provided, the surgical experience and management of the patient. However, regarding literature there is strong evidence that pulmonary outcome is better in thoracic surgery if patients have epidurals [5]. We cannot confirm these data from 1998 either. We explain our good results with the consequent analgesic protocols for both groups until dismission from hospital and our intensive monitoring of pain therapy until dismisssion. To our knowledge, we first described an “aggressive” systemic analgesic regimen in patients undergoing thoracotomy [10],[11].

More male than female patients underwent a pleurectomy. This is most likely due to the fact that the pleura mesothelioma is diagnosed more often in male patients. We assume that this difference in demographic data do not compromise our results.

Our results regarding pre-existing pain therapy and the choose of the analgesic regimens are quite inhomogeously. In all 1555 thoracotomies more patients in the Opioid Group had a preexisting pain therapy than in the EDA Group. Regarding the bilobectomy procedure more patients in the EDA Group had a pre-existing pain therapy than patients in the Opioid Group and regarding the lobectomy procedure, more patients in the Opioid Group than in the EDA Group had a pre-existing pain therapy. One would have expected that more epidurals are placed in patients with pre-existing pain therapy. However, these results underline that our both analgesic protocols are effective. We found that more patients in the EDA Group than in the Opioid Group are dismissed with oral opioid, and when patients are dismissed with opioid medication, patients in the EDA Group are dismissed with higher opioid doses than patients in the Opioid Group. This finding underlines the fact that thoracic surgery needs multimodal analgesic concepts not only in the first postoperative period, but also until dismission from hospital.

We present retrospective data in our observational study. Of course, these data might be biased. We tried to reduce potential biases in patient selection by detailed subgroup analysis. Patients undergoing a metastasectomy e.g. have mostly no compromised lung function, they differ from patients undergoing a lung volume reduction e.g. with chronic obstructive pulmonary disease, mostly GOLD IV with oxygen therapy.

However, there might be a tendency in our department to recommend epidural analgesia to the patients when the lung function is compromised and/or an extended resection of lung parenchyma is planned. More randomized controlled trials with strong inclusion criteria are needed to compare the influence of epidurals on the outcome after thoracic surgery.

## Conclusion

We assessed the protocols of epidural analgesia and systemic analgesia retrospectively in 1555 thoracotomies during 2011-2013 with regard to length of postoperative hospital stay, recovery of bowel function and opioid dose on dismission from hospital. We found no superiority of epidural analgesia. We explain our finding with the consequent analgesic protocols for both groups until dismission from hospital. Our findings underline the fact that thoracic surgery needs multimodal analgesic concepts not only in the first postoperative period, but also until dismission from hospital.

## Authors’ contributions

Sk was responsible for the study and drafted the manuscript. CD performed the data collection and preparation of the data base. KE performend the clinical investigations. GW carried out the statistical analysis. GS supported KE in performing the cloinical investigations. TH participated in the design of the study and supported Sk in drafting the manuscript. All authors read and approved the manuscript.

## Abbreviations

EDA: Epidural analgesia
IV: Intravenously
NRS: Numeric rating scale
POD: Postoperative day
